# Description of *Aegialoalaimus bratteni* sp. n. from Skagerrak and a review of the genus (Aegialoalaimidae, Nematoda incertae sedis)

**DOI:** 10.3897/BDJ.3.e5738

**Published:** 2015-09-03

**Authors:** Oleksandr Holovachov

**Affiliations:** ‡ Swedish Museum of Natural History, Stockholm, Sweden

**Keywords:** Aegialoalaimidae, Bratten, new species, revision, Skagerrak, Sweden, taxonomy

## Abstract

**Background:**

The genus *Aegialoalaimus* de Man, 1907 includes 12 nominal species, of which three species are considered valid, two species were transferred to other genera and seven species have uncertain taxonomic status.

**New information:**

New species, *Aegialoalaimus bratteni* sp. n. was found in Skagerrak off the west coast of Sweden. It is particularly characterized by the 1.5-1.8 mm long body, short papilliform cephalic sensilla, excretory pore opening just posterior to nerve ring level, spicules that are straight in shape, supplements and gubernaculum absent, separating it from other valid species of the genus. It can be further differentiated from *Aegialoalaimus elegans* in having longer body (1.5-1.8 mm in *A. bratteni* sp. n. *vs* 0.8-1.3 mm in *A. elegans*), shape and size of spicules (straight and 22-29 µm long in *A. bratteni* sp. n. *vs* arcuate and 34 µm long in *A. elegans*), absence of precloacal supplements (*vs* seven-eight in *A. elegans*), absence of gubernaculum (*vs* present in *A. elegans*); from *A. setosa* in having shorter tail (c´=2.6-3.1 in *A. bratteni* sp. n. *vs* c´=4.2 in *A. setosa*), shorter cephalic sensilla (0.5-1.0 µm in *A. bratteni* sp. n. *vs* 9 µm in *A. setosa*), shape and size of spicules (straight and 22-29 µm long in *A. bratteni* sp. n. *vs* arcuate and 40-45 µm long in *A. setosa*), absence of precloacal supplements (*vs* eight in *A. setosa*), absence of gubernaculum (*vs* present in *A. setosa*); from *A. leptosoma* in having longer body (1.5-1.8 mm in *A. bratteni* sp. n. *vs* 0.5-0.7 mm in *A. leptosoma*) and other measurements, shape of spicules (straight in *A. bratteni* sp. n. *vs* arcuate in *A. leptosoma*), absence of precloacal supplements (*vs* three-five in *A. leptosoma*), absence of gubernaculum (*vs* present in *A. leptosoma*). Type specimens of *Aegialoalaimus cylindricauda* Allgén, 1933 and *A. paratenuicaudatus* Allgén, 1959 are redescribed and taxonomic status of these two species is re-evaluated. A taxonomic review, tabular compendium and identification key for species of the genus *Aegialoalaimus* are also given.

## Introduction

The genus *Aegialoalaimus* de Man, 1907 was originally described based on single species *Aegialoalaimus elegans* de Man, 1907 from the North Sea (de Man 1907). Subsequently, eleven more species were described within this genus, but only three were considered valid in the most recent review of the genus by Tchesunov (1990). *Aegialoalaimus brevicaudatus* Kreis, 1924 was transferred to the genus *Paraterschellingia*, and *A. amphibulbosus* Gerlach, 1955 – to *Setoplectus*. Two species, *A. setosa* Bouwman, 1981 and *A. leptosoma* Gagarin, 2012, are described well enough to warrant status of valid species (Bouwman 1981, Gagarin 2012), while the status of remaining seven species remains questionable. In this paper a new species of *Aegialoalaimus*, *A. bratteni* sp. n., is described from the bottom sediments collected in the Bratten area (Swedish economic zone of Skagerrak) off the west coast of Sweden.

## Materials and methods

Bottom sediment samples were collected in several locations in the southern part of the Skagerrak designated as Bratten area. All samples were collected with the bottom dredge and further sieved in the lab before fixation. Nematodes were extracted from samples using a decanting and sieving method (smallest mesh sizes: 45 µm or 70 µm). Fresh water was used during sieving to induce osmotic shock in nematodes so they will detach from the substrate. Samples were immediately fixed in 4% formaldehyde. Formaldehyde-preserved specimens were transferred to pure glycerine using Seinhorst (1959) rapid method as modified by De Grisse (1969). Permanent nematode mounts on glass slides were prepared using the paraffin wax ring method. All curved structures were measured along the curved median line.

Abbreviations used in the text are as follows: L = overall body length; a = body length / greatest body diameter; b = body length / pharyngeal region length; c = body length / tail length; c' = tail length / cloacal body diameter; T = length of main gonad (excluding flexures) / body length (expressed in %).

Type specimens of *Aegialoalaimus cylindricauda* Allgén, 1933 (SMNH Type-3559) and *A. paratenuicaudatus* Allgén, 1959 (SMNH Type-8762) from the invertebrate collection of the Swedish Museum of Natural History (SMNH) were used in this study. Despite considerable search efforts, type material of the following species cannot be located in the collection of C. Allgén: *A. conicaudatus* Allgén, 1959, *A. punctatus* (Allgén, 1929), *A. sabulicola* Allgén, 1933 and *A. tenuicaudatus* Allgén, 1932.

## Taxon treatments

### Aegialoalaimus bratteni

Holovachov sp. n.

urn:lsid:zoobank.org:act:FD17D2BA-920B-409F-8DAD-5847310DCFF7

#### Materials

- **Type status:** Holotype. **Location:** waterBody: Skagerrak; country: Sweden; verbatimDepth: 352–374 m; verbatimLatitude: N 58° 19' 15.6''–20.9''; verbatimLongitude: E 10° 29' 33.5''–34.0''; **Identification:** identifiedBy: O. Holovachov; **Event:** year: 2012; month: October; day: 10; habitat: soft bottom; **Record Level:** datasetID: SMNH Type–8763; institutionCode: Department of Zoology, Swedish Museum of Natural History; collectionCode: invertebrate type collection
- **Type status:** Paratype. **Location:** waterBody: Skagerrak; country: Sweden; verbatimDepth: 352–374 m; verbatimLatitude: N 58° 19' 15.6''–20.9''; verbatimLongitude: E 10° 29' 33.5''–34.0''; **Identification:** identifiedBy: O. Holovachov; **Event:** year: 2012; month: October; day: 10; habitat: soft bottom; **Record Level:** datasetID: SMNH Type–8763; institutionCode: Department of Zoology, Swedish Museum of Natural History; collectionCode: invertebrate type collection
- **Type status:** Other material. **Location:** waterBody: Skagerrak; country: Sweden; verbatimDepth: 232–240 m; verbatimLatitude: N 58° 27' 36.7''–43.3''; verbatimLongitude: E 10° 32' 52.0''–59.4''; **Identification:** identifiedBy: O. Holovachov; **Event:** year: 2012; month: October; day: 12; habitat: soft bottom; **Record Level:** datasetID: #147072; institutionCode: Department of Zoology, Swedish Museum of Natural History; collectionCode: general invertebrate collection

#### Description

**Measurements.** Male holotype: L=1754 µm, a=38.1, b=9.6, c=15.4, c´=2.9, T=62.8%. Male paratype: L=1733 µm, a=35.4, b=9.2; c=13.8, c´=3.1, T=54.9%. Additional male: L=1492 µm, a=?, b=8.6, c=13.0, c´=2.6, T=53.8%.

**Figure 1.**
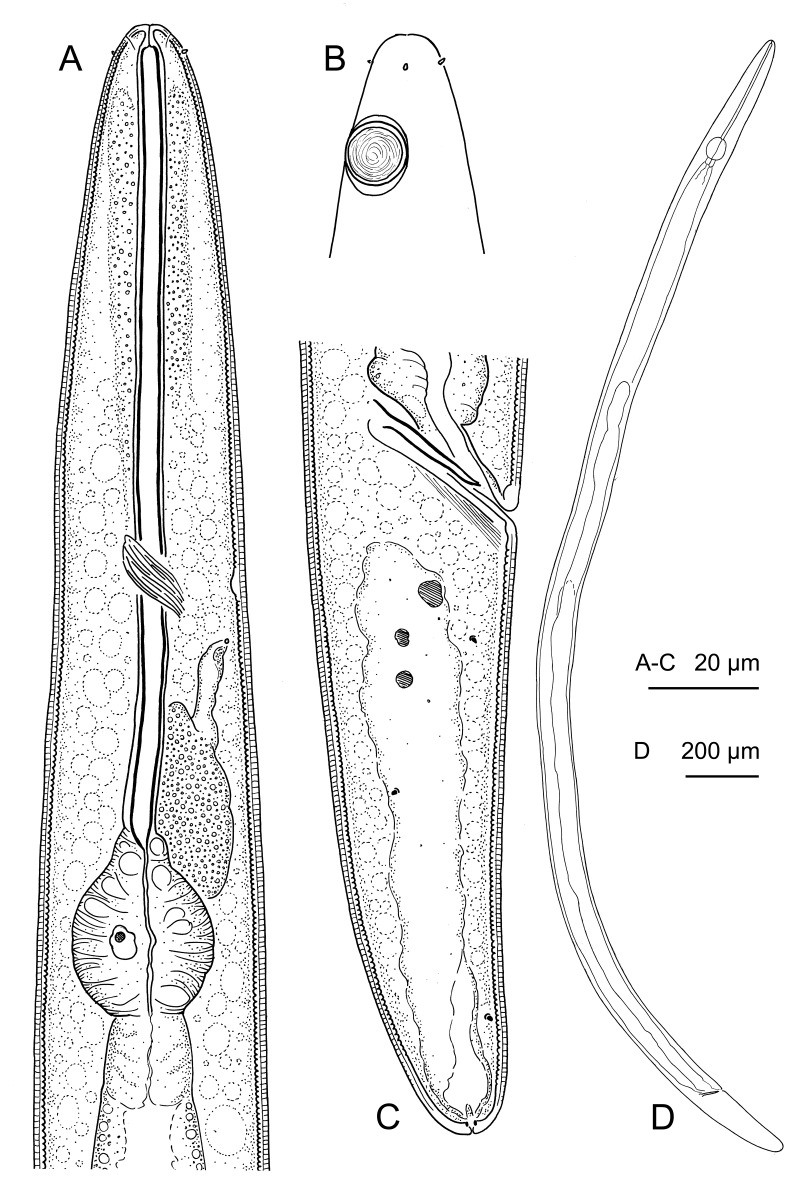
*Aegialoalaimus bratteni* sp.n. from Skagerrak, Sweden. A: Pharyngeal region, median section; B: Anterior body end, surface view; C: Male posterior body region; D: Entire view.

**Male.** (Fig. 1) Body slender, cylindrical over most of its length, tapering anteriorly in the anterior half of pharyngeal region and posteriorly on tail; usually straight or weakly ventrally curved upon fixation; maximum body diameter is 46-49 µm. Cuticle weakly annulated externally, but with distinct transverse striation of inner layers; annules are 1 µm wide, without external ornamentation. Lateral field absent. Crystalloids absent. Body pores and epidermal glands absent. Somatic sensilla present, small papilliform in shape, most distinct along the tail. Labial region rounded, continuous with the body contour, 12.5-13.0 µm wide; lips fused. Inner labial sensilla indistinct. Outer labial sensill pore-like, their nerve endings are distinct. Cephalic sensilla small papilliform, 0.5-1.0 µm long; their bases are located 5-6 µm from the anterior body end. Subcephalic and cervical sensilla absent. Amphidial fovea circular, 10.0-10.5 µm in diameter, with distinct sclerotized margin; its anterior end is located posterior to cephalic sensilla bases, 14-16 µm from the anterior body end. Ocelli absent.

Pharyngeal region is 174-189 m long. Nerve ring surrounds the pharynx at 55.0-58.2% of its length. Hemizonid located at the level with the nerve ring. Secretory-excretory system present; renette cell located on ventral and ventrosublateral sides of the body along the posterior part of pharynx; it extends anteriorly and forms small excretory ampulla just posterior to the nerve ring level. Excretory canal absent, excretory ampulla opens to the exterior on ventral side of the body, at the level with the nerve ring, at 52.9-66.6% of the pharyngeal region length.

Oral opening terminal. Buccal cavity is weakly developed; cheilostom is a narrow tube 3-5 µm long; pharyngostom is indistinguishable from the sclerotized lumen of the pharynx. Pharynx divided into two sections: anterior tubular part and posterior (basal) bulb. Tubular part of the pharynx is 138-151 µm long and 3-4 µm wide; it is surrounded by thin sheath of tissue. Basal bulb is strongly muscularized; 30-33 µm long and 24-28 m wide. Nucleus of the dorsal pharyngeal gland is visible in the middle of the dorsal sector of the basal bulb. Subventral pharyngeal glands indistinct. Pharyngeal gland orifices indistinct. Cardia is 15-18 µm long, its posterior part is embedded in the intestine.

Reproductive system is diorchic, both anterior and posterior testes are outstretched anteriad. Spicules are 20-29 µm long, paired and symmetrical, straight in shape; with conoid shaft and small funnel-shaped manubrium. Gubernaculum absent. Supplements absent. Tail is 114-126 µm long, conoid to subcylindrical in shape, straight or weakly curved ventrad; with bluntly rounded terminus. Caudal glands present, they open to the exterior through common spinneret. Caudal gland nuclei are incaudal.

**Female.** Not found.

#### Diagnosis

*Aegialoalaimus bratteni* sp. n. is particularly characterized by 1.5-1.8 mm long body, short papilliform cephalic sensilla, excretory pore opening just posterior to the nerve ring level, straight spicules, supplements and gubernaculum absent.

#### Taxon discussion

The new species can be differentiated from:

*Aegialoalaimus elegans* (as described by de Man 1907, Schuurmans Stekhoven 1931, Boucher and Helléouët 1977, Jensen 1978b, Bresslau and Schuurmans-Stekhoven 1940, Tchesunov 1990) in having longer body (1.5-1.8 mm in *A. bratteni* sp. n. *vs* 0.8-1.3 mm in *A. elegans*), shape and size of spicules (straight and 22-29 µm long in *A. bratteni* sp. n. *vs* arcuate and 34 µm long in *A. elegans*), absence of precloacal supplements (*vs* seven-eight in *A. elegans*), absence of gubernaculum (*vs* present in *A. elegans*);

*A. setosa* (as described by Bouwman 1981, Holovachov 2014) in having shorter tail (c´=2.6-3.1 in *A. bratteni* sp. n. *vs* c´=4.2 in *A. setosa*), shorter cephalic sensilla (0.5-1.0 µm in *A. bratteni* sp. n. *vs* 9 µm in *A. setosa*), shape and size of spicules (straight and 22-29 µm long in *A. bratteni* sp. n. *vs* arcuate and 40-45 µm long in *A. setosa*), absence of precloacal supplements (*vs* eight in *A. setosa*), absence of gubernaculum (*vs* present in *A. setosa*);

*A. leptosoma* (as described by Gagarin 2012) in having longer body (1.5-1.8 mm in *A. bratteni* sp. n. *vs* 0.5-0.7 mm in *A. leptosoma*) and other measurements, shape of spicules (straight in *A. bratteni* sp. n. *vs* arcuate in *A. leptosoma*), absence of precloacal supplements (*vs* three-five in *A. leptosoma*), absence of gubernaculum (*vs* present in *A. leptosoma*).

Further characters separating all species of the genus are listed in Table 1.

### Aegialoalaimus cylindricauda

Allgén, 1933

#### Materials

- **Type status:** Holotype. **Occurrence:** catalogNumber: SMNH Type-3559; sex: female; **Record Level:** institutionID: Department of Zoology, Swedish Museum of Natural History; collectionID: invertebrate type collection

#### Description

The only type specimen is preserved but its internal structures are poorly visible.

#### Taxon discussion

The original description is based on single female specimen (Allgén 1933: 62-63, Fig. 35). In general morphology, this specimen resembles members of the family Microlaimidae (Fig. 2A): amphid circular in shape; stoma funnel-shaped with small dorsal tooth; pharynx is muscular along its entire length, cylindrical in its anterior part and with well developed basal bulb; female reproductive system didelphic, gonads outstretched. This species is considered *species inquirenda et incerta sedis* within the family Microlaimidae.

### Aegialoalaimus paratenuicaudatus

Allgén, 1959

#### Materials

- **Type status:** Holotype. **Occurrence:** catalogNumber: SMNH Type-8762; sex: female; **Record Level:** institutionID: Department of Zoology, Swedish Museum of Natural History; collectionID: invertebrate type collection

#### Description

The only type specimen is poorly preserved, its anterior end is dried out.

#### Taxon discussion

The original description is based on single female specimen (Allgén 1959: 132, Fig. 134). In general morphology, this specimen resembles members of the family Comesomatidae (Fig. 2B): amphid is multispiral; pharynx is muscular along its entire length, cylindrical in its anterior part and with weak basal swelling; female reproductive system didelphic, gonads outstretched. This species is considered *species inquirenda et incerta sedis* within the family Comesomatidae.

## Identification Keys

### Dichotomous key to species of *Aegialoalaimus* de Man, 1907

**Table:** 

1	Cephalic setae long, 9 µm or equal to 1/2 of the corresponding body diameter	*A. setosa*
–	Cephalic setae short, 1-6 µm long, less than 1/3 of the corresponding body diameter2	2
2	Spicules straight, gubernaculum and supplements absent	*A. bratteni* sp. n.
–	Spicules arcuate, gubernaculum and supplements present	3
3	Male with 3-5 precloacal supplements	*A. leptosoma*
–	Male with 7-8 precloacal supplements	*A. elegans*

## Analysis

### Diagnosis of the genus *Aegialoalaimus* de Man, 1907

*= Tubuligula* Boucher & Helléuët, 1977 op. Jensen, 1978

Cuticle smooth or finely annulated. Lateral alae absent. Epidermal glands absent. Somatic sensilla present. Labial region rounded, continuous with body contour. Inner labial sensilla indistinct. Outer labial sensilla pore-like or papilliform, located on outer surface of lips. Cephalic sensilla papilliform or setiform, located at base of labial region, anterior to amphid. Amphidial aperture circular. Subcephalic and cervical sensilla, deirid and ocelli absent. Secretory-excretory system present; renette cell located opposite to ventral side of pharynx, cardia or anterior part of intestine. Excretory ampulla present. Excretory duct very short, opens to exterior at level with cephalic setae in females and at level of nerve ring in males. Stoma small, undifferentiated. Pharynx divided into anterior long tubular section with strongly sclerotized lumen and weakly developed tissue, and strongly muscularized oval basal bulb. Cardia cylindrical or conoid, partly enveloped by intestinal tissue in its posterior part. Female reproductive system didelphic, amphidelphic, ovary branches reflexed antidromously. Spermatheca present, axial. Vulva equatorial, transverse. Vagina thick; *pars refringens vaginae* absent. Male reproductive system diorchic, both testes outstretched. Spicules symmetrical, straight or arcuate; gubernaculum present or absent. Copulatory apparatus composed of a row of midventral precloacal sensilla (absent in one species). Postcloacal sensilla absent. Tail similar between sexes, conoid or subcylindrical with rounded terminus. Three caudal glands present, open via common spinneret.

### Type species

*Aegialoalaimus elegans* de Man, 1907 (type by monotypy).

= *Tubuligula roscoffensis* Boucher & Helléuët, 1977 op. Jensen, 1978

nec *Aegialoalaimus elegans* sensu Bussau, 1993

### Other valid species

*Aegialoalaimus setosa* Bouwman, 1981

*Aegialoalaimus leptosoma* Gagarin, 2012

*Aegialoalaimus bratteni* sp. n.

### Species inquirendae et incertae sedis

*Aegialoalaimus conicaudatus* Allgén, 1959

*Aegialoalaimus cylindricauda* Allgén, 1933

*Aegialoalaimus paratenuicaudatus* Allgén, 1959

*Aegialoalaimus punctatus* (Allgén, 1929)

= *Kreisia punctata* Allgén, 1929

*Aegialoalaimus sabulicola* Allgén, 1933

*Aegialoalaimus tenuicaudatus* Allgén, 1932

*Aegialoalaimus tenuis* Kreis, 1928

### Species transferred to other genera

*Aegialoalaimus amphibulbosus* Gerlach, 1955 *– Setoplectus amphibulbosus* (Gerlach, 1955)

*Aegialoalaimus brevicaudatus* Kreiss, 1924 – *Paraterschellingia brevicaudata* (Kreiss, 1924)

## Discussion

### *Aegialoalaimus conicaudatus* Allgén, 1959

Type material of this species could not be located in the collection of C. Allgén. Description and illustrations of this species do not provide sufficient information to be able to assign this species to any nematode genus or family (Allgén 1959: 132, Fig. 135a-b). As drawn, pharynx appears to be muscularized along its entire length, cylindrical in its anterior part, with basal bulb; it can match a number of nematode families, but is dissimilar to the pharynx of *Aegialoalaimus*. This species is considered *species inquirenda et incerta sedis* within Nematoda.

### *Aegialoalaimus elegans* sensu Bussau, 1993

Population of *Aegialoalaimus elegans* described by Bussau (1993) differs from the typical *A. elegans* in having shorter body (0.4-0.6 mm in Bussau specimens *vs* 0.8-1.3 in *A. elegans*), shorter spicules (15 µm in Bussau male *vs* 34 µm in *A. elegans*), presence of a small notch on the ventral side of spicules (*vs* absent in *A. elegans*). It is possible that specimens found and described by Bussau (1993) belong to a different, new species of *Aegialoalaimus*, but without examining actual specimens I refrain from naming it.

### *Aegialoalaimus punctatus* (Allgén, 1929)

*Type material of this species could not be located in the collection of C. Allgén. *Aegialoalaimus punctatus* (Allgén, 1929) was originally described as *Kreisia punctata* Allgén, 1929. Description and illustrations of this species do not provide sufficient information to be able to assign this species to any nematode genus or family (Allgén 1929: 461, Fig. 21). As drawn, pharynx appears to be muscularized along its entire length, cylindrical in its anterior part, with basal bulb; it can match a number of nematode families, but is dissimilar to the pharynx of *Aegialoalaimus*. There are two separate circles of setae and the amphid is multispiral – two more characters not found in the genus *Aegialoalaimus*. This species is considered *species inquirenda et incerta sedis* within Nematoda.*

### *Aegialoalaimus sabulicola* Allgén, 1933

Type material of this species could not be located in the collection of C. Allgén. Description and illustrations of this species do not provide sufficient information to be able to assign this species to any nematode genus or family (Allgén 1933: 64-65, Fig. 37). As drawn, tail appears to be conoid with well developed spinneret and is dissimilar to tail shape typical for valid species of the genus *Aegialoalaimus*. This species is considered *species inquirenda et incerta sedis* within Nematoda.

### *Aegialoalaimus tenuicaudatus* Allgén, 1932

Type material of this species could not be located in the collection of C. Allgén. Description and illustrations of this species do not provide sufficient information to be able to assign this species to any nematode genus or family (Allgén 1932: 414-415, Fig. 5). As drawn, pharynx appears to be muscularized along its entire length, cylindrical in its anterior part, with basal bulb; it can match a number of nematode families, but is dissimilar to pharynx of *Aegialoalaimus*. Similarly, population described as *A. tenuicaudatus* by Allgén (Allgén 1933: 63-64, Fig. 36) does not match the diagnosis of *Aegialoalaimus*. On the other hand, both show superficial resemblance to the family Microlaimidae in the shape of the amphid, pharynx and tail. Subsequent redescription of *A. tenuicaudatus* by Allgén (1935) was positively identified as *Molgolaimus turgifrons* Lorenzen, 1972 by Jensen (1978a). Therefore, we consider this species to belong to the family Microlaimidae.

### *Aegialoalaimus tenuis* Kreis, 1928

*Aegialoalaimus tenuis* Kreis, 1928 most probably should be placed in the family Microlaimidae based on the following characters: pharynx cylindrical in its anterior part, with basal bulb, muscularized along its entire length; reproductive system didelphic, gonads outstretched (Kreis 1928).

## Supplementary Material

XML Treatment for Aegialoalaimus bratteni

XML Treatment for Aegialoalaimus cylindricauda

XML Treatment for Aegialoalaimus paratenuicaudatus

## Figures and Tables

**Figure 2a.**
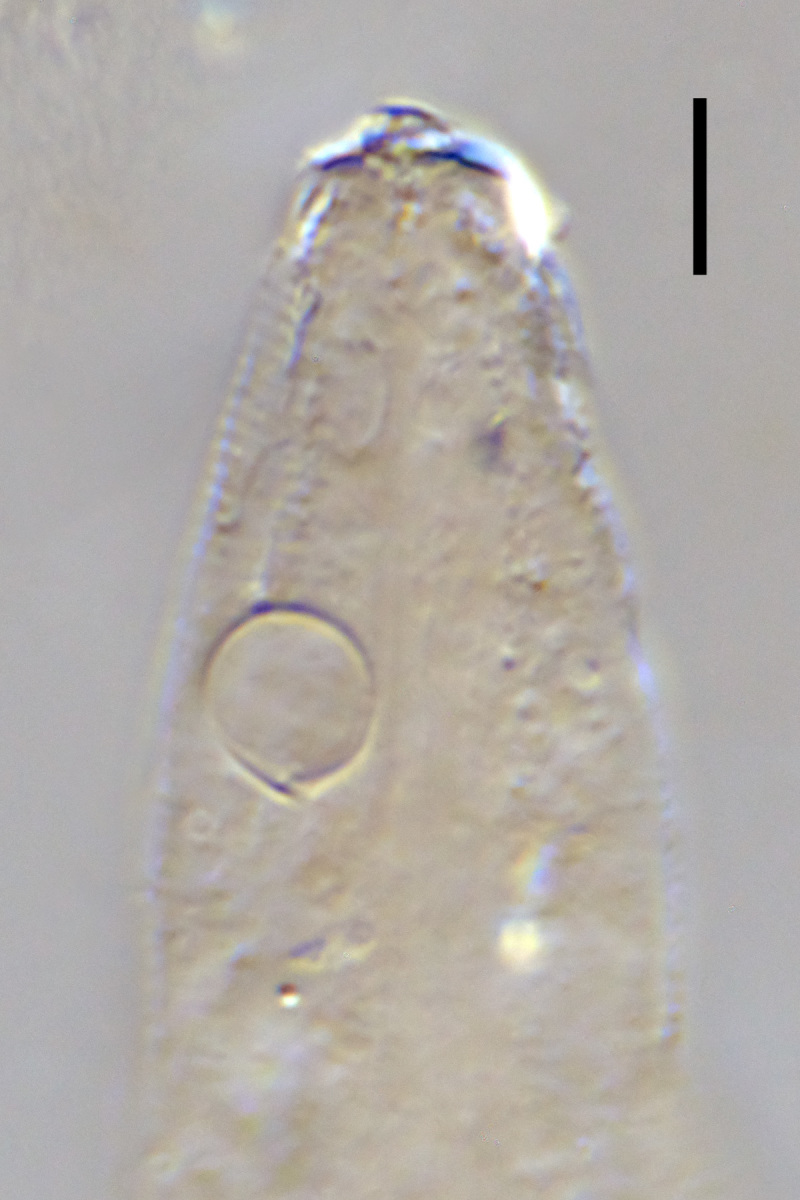
*Aegialoalaimus cylindricauda* Allgén, 1933.

**Figure 2b.**
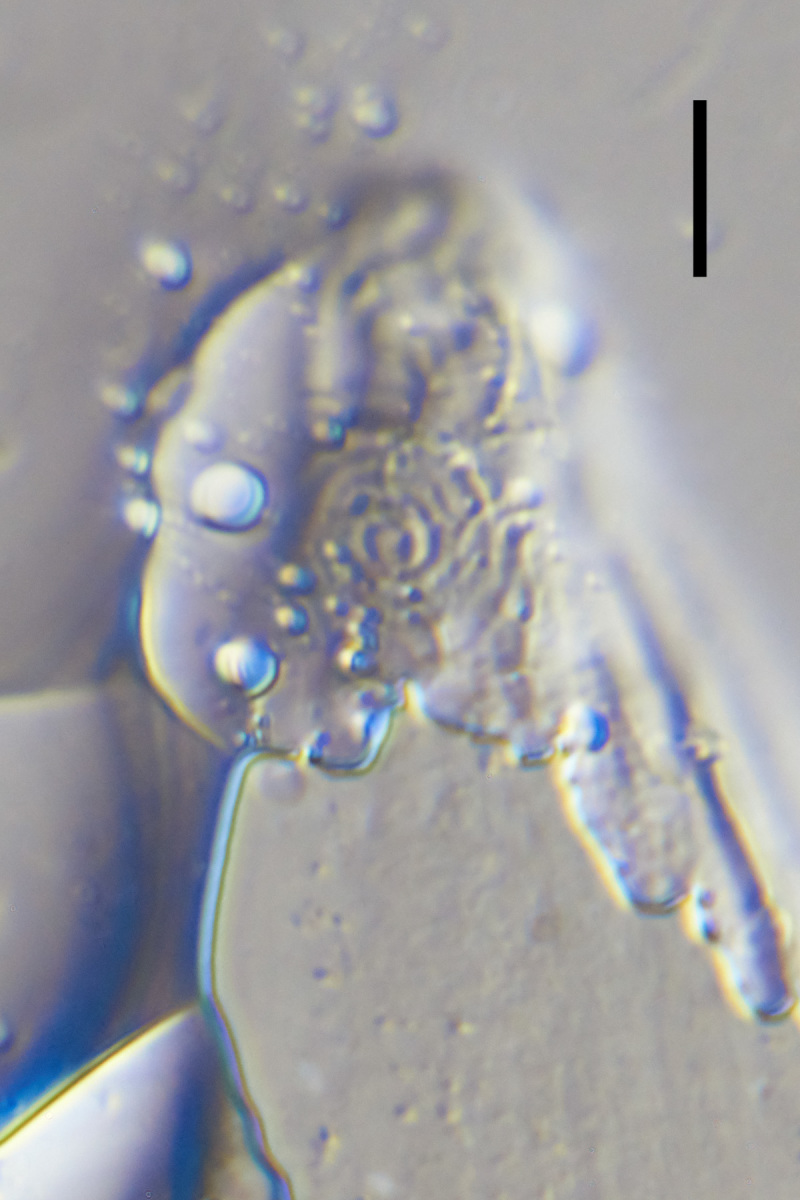
*Aegialoalaimus paratenuicaudatus* Allgén, 1959.

**Table 1.** Diagnostic characters of valid species of the genus *Aegialoalaimus* de Man, 1907 and *A. elegans* sensu Bussau (1993) based on recent observations and literature data.

Character	*A. elegans* de Man, 1907	*A. leptosoma* Gagarin, 2012	*A. setosa* Bouwman, 1981	*A. elegans* sensu Bussau 1993	*A. bratteni* sp. n.
Body length (mm)	0.8-1.3	0.5-0.7	1.4-1.8	0.4-0.6	1.5-1.8
Tail length (µm)	81-102	54-68	≈130	45-75	114-126
c'	3.0-5.0	3.1-4.0	5.0-6.0	4.2	2.6-3.1
Cephalic setae length (µm)	1.0-6.0	1.5-2.0	≈9	2.0	0.5-1.0
Spicules length (µm)	34	23-24	40-45	15	22-29
Supplements	7-8	3-5	8	?	absent
Excretory pore in male	nerve ring level	?	nerve ring level	nerve ring level	nerve ring level
Excretory pore in female	cephalic setae bases	?	?	?	NA
Spicule shape	arcuate	arcuate	arcuate with notch	arcuate with notch	straight
Gubernaculum	present	present	present	present	absent
